# Genetic Basis of the Antioxidant and Serum Enzyme Activities of the Large Yellow Croaker *Larimichthys crocea* Under Stress in an Experimental Simulation of Natural Winter Water Cooling

**DOI:** 10.3390/antiox14101260

**Published:** 2025-10-20

**Authors:** Xinan Wang, Xiaolong Yin, Aijun Ma, Weiye Li, Xiaolin Zhang

**Affiliations:** 1 State Key Laboratory of Mariculture Biobreeding and Sustainable Goods, Yellow Sea Fisheries Research Institute, Chinese Academy of Fishery Sciences, Qingdao 266071, China; wangxa@ysfri.ac.cn; 2 China-ASEAN Belt and Road Joint Laboratory on Mariculture Technology (Qingdao), Qingdao 266071, China; 3 Laboratory for Marine Biology and Biotechnology, Qingdao Marine Science and Technology Center, Qingdao 266237, China; 4 Zhoushan Fisheries Research Institute, Zhoushan 316004, China; xlyhndx@163.com; 5 Marine Science and Technology College, Zhejiang Ocean University, Zhoushan 316022, China; zhangxiaolin@zjou.edu.cn

**Keywords:** genetic effects, antioxidant activity, serum enzyme activity, natural water cooling stress, large yellow croaker

## Abstract

Antioxidant and serum enzyme activities of the large yellow croaker (Larimichthys crocea) were analyzed to investigate the influences of genotype and water temperature. The activities of four antioxidant factors (peroxidase, catalase, total antioxidant capacity, and superoxide dismutase [SOD]) in the muscle and liver, as well as six serum enzymes (alkaline phosphatase, lipase, aspartate aminotransferase [AST], adenosine deaminase, γ-glutamyl transpeptidase (GGT), and alanine aminotransferase), were measured at natural water temperatures (20, 16, 12, 10, and 8 °C). Analysis of the antioxidant enzyme activities showed that genotype, temperature, and genotype × temperature interactions had different influences on the two tissues. In muscles, the impacts of these three effects on antioxidant enzyme activity were extremely significant, while in the liver, only the genetic effects were extremely significant and the temperature effect was insignificant. SOD exhibited the highest and the most stable activity and the best performance in terms of both activity and stability in both tissue types. Temperature, genotype, and genotype × temperature interactions all had a prominent impact on serum enzyme activity. The indicators that were the top performers in terms of activity, stability, and the optimal balance of both were AST, GGT, and AST, respectively. Our findings provide a theoretical basis for breeding with low-temperature tolerance based on antioxidant factors, reliable tolerance indicators, and the evaluation and early detection of low-temperature stress using serum-based enzyme biomarkers.

## 1. Introduction

Water temperature is an important environmental factor in aquaculture systems [1,2,3]. It not only affects the growth, development, and survival of aquatic animals but also exerts a remarkable influence on their immune systems [4,5,6]. Challenging temperatures affect aquatic animals by repressing growth and damaging their immune systems, leading to diseases and serious losses in the aquaculture industry [7,8]. Several studies on fish have shown that water temperature has direct effects on fish growth, disease resistance, and survival [9,10,11,12].

The large yellow croaker (*Larimichthys crocea*) is a warm-water fish, largely distributed in the East China Sea, the southern Yellow Sea, and the northern coast of the South China Sea east of the Qiongzhou Strait. It is an important economic species in China. It is adapted to water temperatures in the range of 10–32 °C, with an optimal temperature of 18–25 °C [13]. Feeding decreases at temperatures below 14 °C and above 30 °C, and death occurs at temperatures below 7 °C [14]. Recent climate change events have intensified extreme weather events. In southern China, the coastal areas have also been frequently affected by cold currents, resulting in long-term low water temperatures in aquaculture areas, causing serious damage to large yellow croaker populations that cannot tolerate low temperatures. In 2016, the economic losses caused by a cold wave in the fisheries of southern China exceeded CNY 2 billion, with large yellow croaker losses alone exceeding CNY 300 million [15]. Therefore, research into cold resistance in the large yellow croaker would be of great assistance in developing breeding programs for low-temperature tolerance and the development of low-temperature resistance control measures in aquaculture [15,16] and would promote sustainability in the large yellow croaker aquaculture industry.

Water temperature is a vital factor influencing the physiological activities of fish and other aquatic animals. Abnormal temperatures can directly affect the antioxidant enzyme activity and reactive oxygen species (ROS) content of fish, activating an environmentally adaptive stress response [17]. Under prolonged stress, fish will inevitably suffer a decline in immune defenses and disease resistance, affecting their growth, especially in young fish [18]. Antioxidant enzymes function to eliminate reactive oxygen species and play an important role in enhancing phagocyte activity and immune function [19]. Serum biochemical factors are important indicators reflecting changes in the metabolism and functional status of tissues and organs in animals under environmental stress [20,21]. According to Selye’s stress theory [22], under low-temperature stress, the physiological changes in experimental fish will inevitably be reflected in their blood biochemical factors. Therefore, antioxidant enzymes and serum biochemical factors can act as indicators to evaluate fish breeding programs for tolerance to low temperatures. Investigating the activity of serum biochemical factors, together with antioxidant enzymes, in fish is important for the assessment of their physiological health and environmental adaptability.

Zhang et al. (2013) reported variations in serum and antioxidant enzyme activities in the large yellow croaker at different temperatures during cooling periods in natural sea areas [16]. The liver was shown to play a key role in removing reactive oxygen species and in antioxidant regulation, and water temperature had an impact on serum enzyme activity. However, different activities of serum biochemical factors combined with antioxidant enzymes are probably attributable to not only different water temperatures but also fish genotypes that affect antioxidant enzymes/serum biochemical factors, as well as to genotype × temperature interactions. Therefore, the genetic analysis of antioxidant/serum enzyme activities in the large yellow croaker would be of vital importance for understanding their genetic effects. The genetic analyses of antioxidant enzymes in *Scophthalmus maximus* and *Takifugu rubripes* have been conducted [17,23,24]. However, no similar studies on the large yellow croaker have been reported.

This study explored the genotype × temperature interactions affecting antioxidant enzymes/serum enzymes in the large yellow croaker using genotype main effects plus genotype × environment interaction (GGE) biplot analysis [25] and additive main effects and multiplicative interactions (AMMI) [26]. The results were used to provide a reference to formulate a breeding plan for enhanced low-temperature tolerance in the large yellow croaker.

## 2. Materials and Methods

### 2.1. Materials and Experimental Methods

The experiment was conducted in the winter of 2024 at the Shinuo Marine Aquatic Limited Corporation in Zhoushan, Zhejiang, China. The experimental fish were the offspring of the Dai-ju stock of *Pseudosciaena crocea*, which were raised by the Shinuo Marine Aquatic Limited Corporation. The experimental fish were uniform in size (a mean body length of 24.77 ± 1.94 cm and a mean body mass of 198.75 ± 45.07 g) and cultured in a cage. Artificial floating expanded feed is used to breed the large yellow croaker. Feed was provided to the fish twice a day, once in the morning and once in the evening. The daily feeding amount was 1% to 3% of their body weight. The sampling period was from 7 November 2024 to 16 December 2024. The water temperature in the cage was reduced from 20 °C to 8 °C over a period of 39 days and was measured daily in the morning, at midday, and in the evening. Fish blood samples were collected at 20, 16, 12, 10, and 8 °C. On each sampling date, six fish from each temperature group were randomly taken and anesthetized with MS-222 (tricaine methanesulfonate, supplied by Maya Reagent Limited Corporation, Jiaxing, China). A total of 30 fish were utilized in this study.

In the sampling process, the fish were quickly removed from the water, and 20 µL of their blood was collected from the tail vein using a collection syringe. The samples were then placed into a centrifuge tube. The centrifuge tube was first soaked in a 1% heparin sodium solution and then dried and cooled prior to use. Collected blood samples were left to stand for 5 h in a 4 °C incubator. They were then centrifuged for 15 min at 4 °C at 4000 r/min. The upper serum was removed and frozen for subsequent testing. Simultaneously, muscle samples and the liver were resected, refrigerated using liquid nitrogen, and preserved at −80 °C for subsequent testing.

### 2.2. Sample Analyses

#### 2.2.1. Determination of Antioxidant Factors in the Muscle and Liver Tissues

Normal saline was added to both the muscle and liver samples in a volume that was nine times the sample volume (volume fraction: 0.86%) per gram of tissue. An ice bath with a glass homogenizer was used to homogenize the samples, and the samples were then centrifuged for 30 min at 4 °C at 12,000 r/min. The supernatant was removed for enzyme activity analysis.

The peroxidase (POD), catalase (CAT), and superoxide dismutase (SOD) activities and the total antioxidant capacity (T-AOC) in the muscle and liver samples were measured using kits from the Nanjing Jiancheng Biological Engineering Research Institute (Nanjing, China), following the supplier’s instructions.

The POD activity was determined using a spectrophotometer method. One unit of POD activity (U/mg) is defined as the amount of enzyme that causes the absorbance at 470 nm to increase by 0.01 per minute per milligram of tissue protein in the reaction mixture. The CAT activity was determined by the visible-light spectrophotometric method, with the decrease in H_2_O_2_ monitored at 450 nm. One unit of enzyme activity (U/mg) was defined as the decomposition of 1 μmol of H_2_O_2_ per milligram of tissue protein per second. The SOD activity was determined by the WST-1 assay. Absorbance was measured colorimetrically at 550 nm for activity calculation. One unit of enzyme activity (U/mg) is defined as the amount of SOD required to achieve 50% inhibition in 1 mL reaction mixture per milligram of tissue protein. The T-AOC activity was detected using a spectrophotometer method. One unit of T-AOC activity (U/mg) is defined as an increase in absorbance of 0.01 per minute per milligram of tissue protein, as measured by the colorimetric (spectrophotometric) method.

#### 2.2.2. Determination of Serum Biochemical Factors

Levels of serum alkaline phosphatase (ALP), serum lipase (LIP), aspartate aminotransferase (AST), adenosine deaminase (ADA), γ-glutamyl transpeptidase (GGT), and alanine aminotransferase (ALT) were measured by commercial kits (Nanjing Jiancheng Biological Engineering Research Institute, Nanjing, China) in the indicated experimental groups based on the manufacturer’s instructions.

#### 2.2.3. Data Analysis

##### Split-Plot (SP) Analysis

The experiment was conducted using an SP design, in which temperature was the main-plot factor and six complete replicate blocks had five separate main plots, each assigned to five temperature gradients (20, 16, 12, 10, and 8 °C). Antioxidant factors/serum enzymes were sub-plot factors with four antioxidant factors (SOD, POD, CAT, and T-AOC)/six serum enzymes (ALT, AST, ALP, LIP, ADA, and GGT) distributed in three sub-plots under every main plot. The SP analysis model can be represented by the following equation:

(1) $$y_{mnp}=\mu +a_{p}+g_{mn}+h_{mp}+\varepsilon_{mp}$$

where $y_{mnp}$ denotes the activity of the *n*-th antioxidant enzyme/serum enzyme under the *m*-th temperature treatment in the *p*-th complete block; μ, $a_{p}$, and $g_{mn}$ denote the general intercept, the impact of the *p*-th block, and the *mn*-th treatment effect, respectively;$\;h_{mp}$ denotes the main-plot error in relation to the *m*-th activity gradient and the *p*-th block, with randomization, zero mean, and variance $\sigma_{h}^{2}$; and$\;\varepsilon_{mp}$ denotes the residual sub-plot error possessing variance $\sigma^{2}$ together with zero mean.

##### AMMI Analysis

Principal component analysis combined with analysis of variance was integrated into the AMMI model [26]. For example, the AMMI model for the *g*th genotype (antioxidant factors, including SOD, POD, and CAT, and T-AOC/serum enzymes, including ALT, AST, ALP, LIP, ADA, and GGT) at the *e*th temperature (20, 16, 12, 10, and 8 °C) is as follows:

(2) $$y_{ge}=\mu +\rho_{g}+\psi_{e}+\sum_{i=1}^{M}\theta_{n}\xi_{gn}\eta_{en}+\varepsilon_{ge}$$

where $y_{ge}$ represents the activity of antioxidant factors/serum enzymes at temperature *e* for genotype *g*; $\psi_{e}$ represents the average temperature deviation; μ represents the grand mean; $\rho_{g}\;$represents the average genotype deviation; $\xi_{gn}$ and $\eta_{en}$ represent the *n*th principal component about the principal component scores of genotype and temperature, respectively; $\theta_{n}$, *M*, and$\;\varepsilon_{ge}$ represent the eigenvalue of the *n*th interaction principal component axis, the total number of principal component axes, and the residual, respectively.

##### GGE Biplot Analysis

The complicated interactions of the various factors can be clarified using GGE biplot analysis [27,28,29]. Regarding the antioxidant factors/serum enzyme activities detected at varying temperatures, the data were organized into a two-way table comprising the activities of the antioxidant factors/serum enzymes and temperature. An antioxidant factor/serum enzyme was allocated to each mean value of activity under the matched temperature. The top two principal components were subjected to the decomposition of singular value to fit the GGE biplot model [25] as follows:

(3) $$y_{ge}=\mu +\psi_{e}+\theta_{1}\xi_{g1}\eta_{e1}+\theta_{2}\xi_{g2}\eta_{e2}+\varepsilon_{ge}$$

where $y_{ge}$ denotes the mean trait activity of genotype *g* at temperature *e*; μ, $\psi_{e}$, and $\mu +\psi_{e}$ denote the gross average, major impact of temperature *e*, and average activity among diversified genotypes under temperature *e*, respectively; $\xi_{g1}$ and $\xi_{g2}$ denote the genotype *g* eigenvectors for PC1 and PC2, respectively; $\theta_{1}$ and $\theta_{2}$ denote the PC1 and PC2 values; $\eta_{e1}$ and $\eta_{e2}$ denote the eigenvectors of temperature *e* associated with PC1 and PC2, respectively; and $\varepsilon_{ge}$ denotes the residual in relation to genotype *g* under temperature *e*.

A DPS Data Processing System (Fourth Edition, Hangzhou Ruifeng Information Technology Co., Ltd., Hangzhou, China) [30] was used to complete the AMMI, SP, and GGE biplot analyses.

## 3. Results

### 3.1. SP Analysis of Variance

#### 3.1.1. SP Analysis of Variance of Antioxidant Activities in the Liver Tissue Under Stress from Natural Water Cooling

Table 1 shows the results of the SP analysis of variance for the low-temperature resistance experiment. The *p-*values were 0.2217 for temperature, <0.001 for antioxidant factor, and 0.00456 for the temperature × antioxidant interactions, indicating that the temperature × antioxidant interactions and antioxidant factor had significant (*p* < 0.01) impacts on the activities of the four antioxidant factors, and that the impact of temperature was insignificant (*p* > 0.05).

#### 3.1.2. SP Analysis of Variance of the Antioxidant Activity in the Muscle Tissue Under Stress from Natural Water Cooling

Table 2 shows the SP analysis of variance results. The antioxidant factor, temperature, and the temperature × antioxidant interactions in Table 3 all had *p*-values of <0.001, suggesting that antioxidant factor, temperature, and the temperature × antioxidant interactions had significant (*p* < 0.01) effects on the activity of the four antioxidant factors.

#### 3.1.3. SP Analysis of Variance of the Serum Enzyme Activity in the Blood Under Stress from Natural Water Cooling

Table 3 shows the results of the SP analysis of variance of the low-temperature resistance experiments. The serum enzymes, temperature × serum enzymes, and temperature all had *p*-values of <0.001, indicating that the activities of the six serum enzymes were significantly (*p* < 0.01) impacted by temperature, serum enzyme, and temperature × serum enzyme interactions.

### 3.2. AMMI Analysis of Variance

#### 3.2.1. AMMI Analysis of the Antioxidant Activity in the Liver Tissue Under Stress from Natural Water Cooling

The AMMI analysis results under varying temperatures (Table 4) show that genotype had a very significant (*p* < 0.01) influence and that the genotype × temperature interactions had significant, but weaker, impacts on antioxidant factor activities (*p* < 0.05). However, the influence of temperature was not significant (*p >* 0.05). Overall, for the antioxidant factor activities, the total sum of squares (SS) was 94.0799% for the genotype effects, 0.3135% for the temperature effects, and 1.0445% for the genotype × temperature interactions.

#### 3.2.2. AMMI Analysis of the Antioxidant Activity in the Muscle Tissue Under Stress from Natural Water Cooling

Based on the AMMI analysis results, different temperatures, genotypes, and genotype × temperature interactions all had significant (*p* < 0.01) impacts on antioxidant activities. Overall, the genotype, temperature, and genotype × temperature interactions were responsible for 85.2102%, 4.1430%, and 8.4604% of the total SS of the antioxidant factor activities, respectively (Table 5).

#### 3.2.3. Results of the AMMI Analysis of the Serum Enzyme Activity in the Blood Under Stress from Natural Water Cooling

The AMMI analysis showed that genotype × temperature interactions, temperature, and genotype all significantly influenced the activities of serum enzymes (*p* < 0.01) at various temperatures. Overall, the genotype × temperature interactions, genotype, and temperature effects contributed to 89.5598%, 2.0048%, and 6.2543% of total SS of serum enzyme activity, respectively (Table 6).

### 3.3. GGE Biplot Analysis

**Figure 1:**
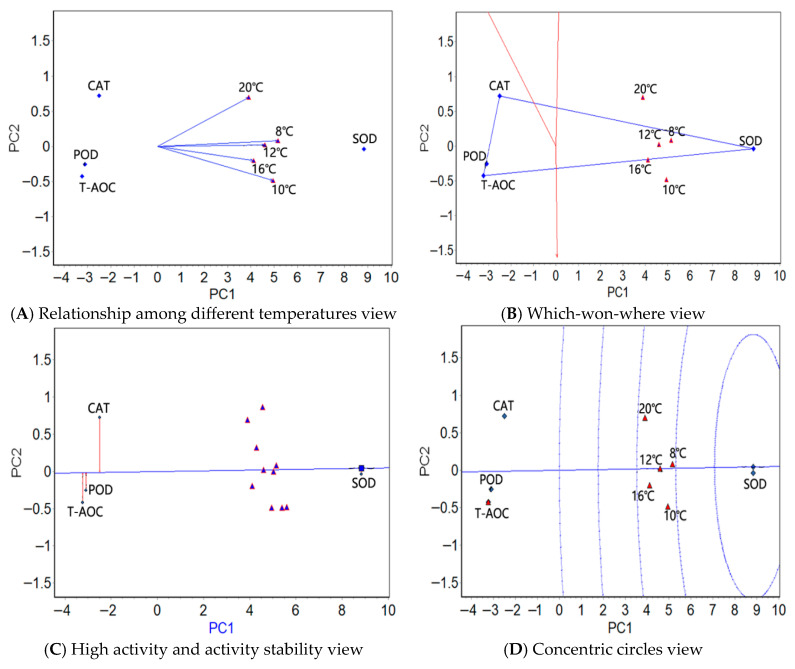
The GGE biplots of the antioxidant factors in the liver tissue of the large yellow croaker under different temperatures.

**Figure 2:**
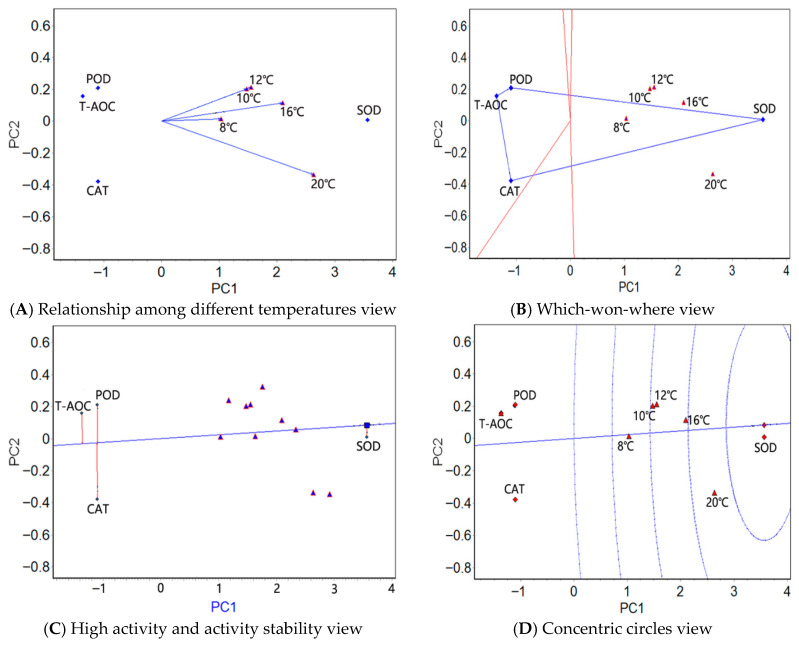
The GGE biplots of the muscle tissue antioxidant factors of the large yellow croaker at different water temperatures.

**Figure 3:**
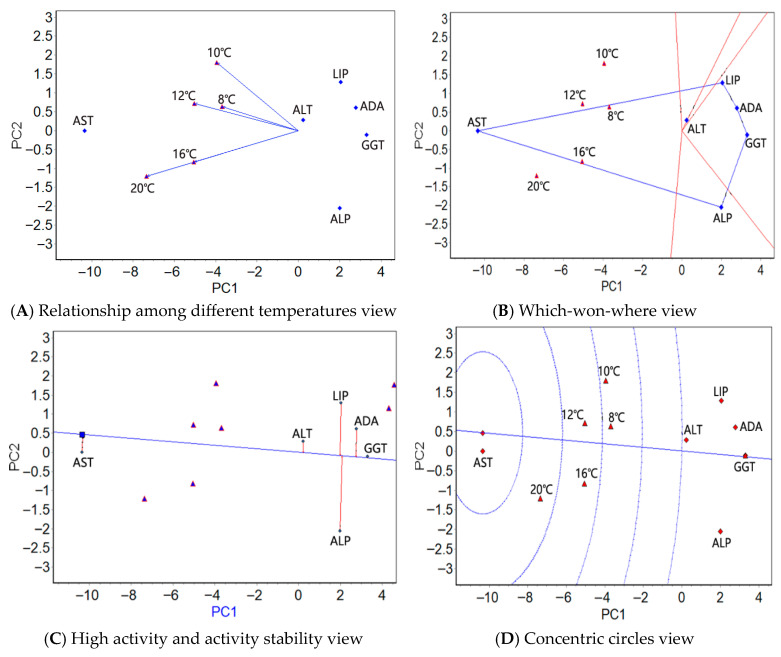
The GGE biplots of the serum enzymes of the large yellow croaker at different water temperatures.

The mean activities of the four antioxidants/six serum enzymes at five temperatures were used in a GGE biplot analysis (Supplementary Materials Section SA: Tables S1–S3). The “relationship among different temperatures” view (panel A in Figure 1, Figure 2 and Figure 3), the “which-won-where” view (panel B in Figure 1, Figure 2 and Figure 3), the “high activity and activity stability” view (panel C in Figure 1, Figure 2 and Figure 3), and the “concentric circles” view (panel D in Figure 1, Figure 2 and Figure 3) were drawn using the GGE biplot analysis results (Supplementary Materials Section SA: Tables S4–S6); see Supplementary Materials Section SB for a detailed explanation of the GGE biplot views.

#### 3.3.1. GGE Biplot Analysis of the Antioxidant Enzymes in the Liver Tissue Under Stress from Natural Water Cooling

In the liver tissue, the relationship among the different temperatures view (Figure 1A) showed the smallest angles (close to zero) in the range 8–12 °C, indicating that the four physiological indexes had almost identical activity rankings between these temperatures. The angles in the range 10 °C to 20 °C were the greatest, indicating that the difference in the activity rankings of the four physiological indicators is the greatest at these two temperatures. The four antioxidant factors were the easiest to distinguish at 10 °C. The five test temperatures in the which-won-where view (Figure 1B) were clustered in one region, where SOD was the most active. The high activity and activity stability view (Figure 1C) showed that SOD was the most active, on average, followed by CAT, POD, and T-AOC; SOD also showed the most stable activity, followed by POD, T-AOC, and CAT. The greatest activity and stability of SOD emerged in the concentric circles view (Figure 1D).

#### 3.3.2. GGE Biplot Analysis of the Antioxidant Enzymes in the Muscle Under Stress from Natural Water Cooling

In the muscle tissue, the angles between 10 °C and 12 °C were zero in the relationship among different temperatures view (Figure 2A), implying that the four physiological indexes had identical activity rankings at these two temperatures. The angles between 20 °C and 10 °C/12 °C were greater, indicating that the differences in the activity rankings of the four physiological indexes were the greatest at these two temperatures. The four antioxidant factors were the easiest to distinguish at 20 °C. Similarly, the five test temperatures were classified as one region in the which-won-where view (Figure 2B), in which SOD showed the highest activity. As in the liver tissue, SOD exhibited the highest average activity in the high activity and activity stability view (Figure 2C), followed by CAT and POD; SOD showed the most stable activity, followed by T-AOC and POD. As in the liver tissue, the highest activity and stability of SOD was illustrated in the concentric circles view (Figure 2D).

#### 3.3.3. GGE Biplot Analysis of the Serum Enzymes in the Blood Under Stress from Natural Water Cooling

In the blood, in the relationship among different temperatures view (Figure 3A), the angles between 16 °C and 20 °C were zero, hinting that the six plasma physiological indexes shared the same activity ranking under these two temperatures. As in the liver and muscle, the angles between 10 °C and 20 °C were greater, indicating that the differences in the activity rankings of the six plasma physiological indexes are the greatest at these two temperatures. The activity of the six plasma physiological indexes was the easiest to distinguish at 20 °C. As in the liver and muscle, the which-won-where view showed one region containing the five test temperatures (Figure 3B) and the highest AST activity. Based on the high activity and activity stability view (Figure 3C), AST showed the greatest average activity, followed by ALT and LIP; GGT, ALT and AST ranked from high to low in terms of stable activity. The activity and stability of AST were the greatest in the concentric circles view (Figure 3D).

## 4. Discussion

### 4.1. Genetic Influence on Antioxidant Enzyme Activity in Two Tissues of Larimichthys crocea

The theory of free radical damage suggests that physiological damage during environmental stress is mainly related to excessive ROS generation [31,32,33,34]. In normal contexts, the antioxidant system can eliminate ROS, preventing their production from reaching damaging levels [35,36,37,38,39,40]. SOD, POD, and CAT are important protective enzymes that play crucial roles in clearing reactive oxygen species. The T-AOC value reflects the capability of the non-enzymatic and antioxidant enzyme systems to compensate for external stimuli and is a good indicator of tissue antioxidant function [41,42,43,44].

The AMMI analysis of the antioxidant activity in the liver and muscle samples showed that, although the ranking of the three effects was the same, i.e., genotype > temperature > genotype × temperature interactions, their effects on antioxidant enzyme activity were not the same in the two tissues. In muscles, the impact of all three effects was extremely significant (*p* < 0.01), while in the liver, only the genetic effect reached a highly significant level (*p* < 0.01), with temperature failing to exert a significant effect (*p* > 0.05). This implies that the three factors affecting antioxidant enzyme activity had similar characteristics and specificities in the two tissues. We speculated that this difference stems from the tissue specificity of antioxidant enzyme activity. Temperature has no significant effect on the liver, possibly because the liver is less involved than the muscles in responding to temperature changes. The liver is not directly affected by the external environment, as muscles are. The AMMI analysis of antioxidant enzyme activities in the liver of *Takifugu rubripes* at different temperatures showed that 93.532%, 2.978%, and 2.026% of the total sum of squares for antioxidant activity were attributable to the genotype effect, temperature effect, and the genotype × temperature interaction, respectively [23]. The AMMI analysis of antioxidant enzyme activities in the liver of *Scophthalmus maximus* at different temperatures showed that 82.4720%, 4.0666%, and 12.0968% of the total sum of squares for antioxidant activity were attributable to the effects of genotype, temperature, and genotype× temperature interaction, respectively [17]. Clearly, the genotype effect is the dominant factor influencing antioxidant enzyme activity in both *T. rubripes* and *S. maximus*, which was consistent with the conclusion of this study. We speculated that this may be due to the lower temperature limit (i.e., in the low-temperature resistance test) being set too high or the upper temperature limit (i.e., in the high-temperature resistance test) being set too low in the temperature stress experiment. In *T. rubripes*, the ranking of the three effects was the genotype effect > the temperature effect > the genotype × temperature interaction [23], and in *S. maximus*, it was the genotype effect > the genotype × temperature interaction > the temperature effect [17]. Obviously, the ranking of the three effects observed in this study is consistent with the findings in *T. rubripes*, whereas it shows minor discrepancies from those in *S. maximus*. This indicated that the relative importance of genotype effects, temperature effects, and genotype temperature interactions on antioxidant enzyme activity is not entirely consistent across different species.

The GGE biplot analysis of the antioxidant activity in the liver and muscle tissues showed that the temperature variations with the closest and farthest activity ranking of the four physiological indexes differed in the two tissues (the temperature group with the farthest activity ranking was between 20 °C and 10/12 °C in the muscle and between 20 °C and 10 °C in the liver). For these two tissues, the temperature that best distinguishes the activity of the four antioxidant factors is not the same. In both tissues, the test temperatures were concentrated in a region in which SOD was the most active enzyme. In addition, SOD showed the highest activity, the most stable activity, and the best activity and stability in both of the two tissues. Therefore, the conclusions drawn from the GGE biplot analysis were similar to those drawn from the AMMI analysis; there are significant similarities in the genetic basis of the antioxidant enzyme activity at different temperatures in the two tissues, as well as some differences. In *T. rubripes* and *S. maximus*, the GGE biplot analysis revealed that SOD exhibited the highest activity and stability under different temperatures [17,23]. Although the antioxidant factors used in current research on the yellow croaker are not exactly the same as those used in *T. rubripes* and *S. maximus*, this finding is in agreement with the conclusion of our study. We speculated that, among antioxidant enzymes, SOD may exhibit a stronger response to temperature stress in fish. In addition, SOD is the first line of defense in the antioxidant system. During the antioxidant process under temperature stress, the activity level of SOD is the first key signal indicating whether the entire process is proceeding effectively [45]. Therefore, the current conclusion may be related to the fact that, in the early stage of temperature stress in fish, the activity response of SOD is usually more rapid and significant than that of POD, CAT, and T-AOC.

Currently, our focus is primarily on the activity of antioxidant enzymes. However, we have not simultaneously assessed the direct molecular damage caused by oxidative stress, such as lipid peroxidation products, protein carbonyls, or DNA oxidative adducts. Therefore, the current conclusion is primarily based on the evaluation of the body’s defense capabilities. In future research, a combined analysis of the aforementioned oxidative damage markers and antioxidant enzyme indicators will be crucial for constructing a more comprehensive oxidative stress assessment system. This approach will also help to further elucidate the genetic basis of antioxidant activity in the large yellow croaker under low-temperature stress.

### 4.2. Genetic Influence on Serum Enzyme Activity in Larimichthys crocea

Serum enzymes are one of the most important indicators in hematology, and their activity is related to the metabolic level and functional status of the corresponding tissues and organs. Changes in serum enzymes can, to some extent, reflect changes in the functional status of tissues and organs [16]. Among the serum enzymes, AST levels reflect cellular lesions in organs such as the liver and heart [46]. ALP participates directly in physiological processes, affecting the transfer and metabolism of phosphate groups, exerting crucial effects on calcium and phosphorus in the body via absorption and metabolism, and maintaining an appropriate calcium–phosphorus ratio [47]. ALT is mainly present in liver cells. Elevated ALT levels in the blood often indicate liver damage and are important indicators for evaluating liver function [48,49,50]. LPS is an enzyme primarily derived from the pancreas [51]. Its function is to hydrolyze glycerides containing long-chain fatty acids. GGT and ADA mainly originate from the liver and can serve as indicators of liver parenchymal damage [16].

The AMMI analysis of the serum enzyme activity showed that the ranking of the effects of temperature and genetic characteristics on serum enzymes, as well as the interaction between these two factors, was the same as that for antioxidant enzymes in the liver and muscle, with the genetic effect being the highest and the temperature effect the lowest. The extent of the impact of the three effects on serum enzymes was the same as that on antioxidant enzymes in muscles, with all three effects being extremely significant (*p* < 0.01). However, the degree of their influence on antioxidant enzymes in the liver was different, indicating that the influences of the three effects on serum enzymes were similar to their effects on antioxidant enzymes in muscles but significantly different from their effects on antioxidant enzymes in the liver. The AMMI analysis of serum biochemical indexes of turbot at different temperatures and exposure times showed that, at each exposure time, the genotype effect > the genotype × temperature interaction > the temperature effect [24]. This finding is in agreement with the conclusion of this study. Given that the genotype × temperature interaction had a greater effect than temperature alone, we speculated that analyzing this interaction is crucial when studying the effects of temperature on fish serum biochemical indexes.

The GGE biplot analysis of serum enzymes indicates that the two temperatures exhibiting the closest ranking in serum enzyme activity and those exhibiting the closest ranking in antioxidant enzyme activity are different, whereas the two temperatures with the greatest ranking difference are similar. The temperature with the strongest ability to distinguish serum enzymes was different from that at which antioxidant enzymes could be distinguished (12 °C for antioxidant enzymes and 20 °C for serum enzymes). As in the liver and muscle, one region was identified containing all of the test temperatures, in which the highest AST activity was observed. Unlike the antioxidant enzymes in tissues, the three indicators with the highest activity, the most stable activity, and the best activity and stability were AST, GGT, and AST, respectively, and their activities differed. Obviously, the best activity and stability are mainly determined by the highest activity. Under low stress, the activity of AST in fish blood varies among species [16,52]. Therefore, the current conclusion should only be attributed to the characteristics of serum enzymes in the large yellow croaker under low temperature stress.

Clearly, the conclusions of the serum enzyme GGE biplot analysis were similar to those of the AMMI analysis. Although there was some similarity between the genetic basis of serum and antioxidant enzyme activities, the difference between the genetic basis of the two enzyme activities was significantly greater than the difference in the genetic foundation for antioxidant enzyme activities between the two tissues.

## 5. Conclusions

The genetic basis of the antioxidant enzyme activity in *Larimichthys crocea* in the liver and muscle tissues showed that the influences of the three studied factors differed between the two tissues. In muscles, the impact of the three effects was extremely significant, while in the liver, only the genetic influence reached an extremely significant level, and the effect of temperature was insignificant. This study provides lessons for future studies on breeding considering low-temperature tolerance of the large yellow croaker. Based on antioxidant parameters, muscles should be the prime target tissue, rather than the liver, for evaluating antioxidant capacity under low-temperature stress. SOD showed the highest activity, the most stable activity, and the best performance in terms of both activity and stability indicators across the two tissue types. For breeding programs, SOD is the most optimal indicator among the candidate antioxidant factors evaluated in this study.

In *Larimichthys crocea*, the effects of genotype × temperature interactions, temperature, and genotype all have evident impacts on serum enzyme activity. Among them, AST demonstrated the best activity and stability. This suggests that serum enzymes are reliable biomarkers for assessing low-temperature stress or for acting as early-warning indicators in the large yellow croaker, with AST being the most optimal candidate.

## Figures and Tables

**Table 1:** The split-plot analysis of variance of the four antioxidant enzymes in the liver tissue of the large yellow croaker at five temperature gradient points, resulting from stress due to natural water cooling.

Source of Variation	Sum of Square	Degrees of Freedom	Mean Square	*F*-Value	*p*-Value
Blocks (replicates)	106.2773	5	21.2555		
Temperature	215.4319	4	53.858	1.567	0.2217
Main-plot error	687.4302	20	34.3715		
Antioxidant factor	64,644.578	3	21,548.193	690.332 **	<0.001
Temperature × antioxidant factor	717.6689	12	59.8057	1.916 *	0.0456
Split-plot error	2341.067	75	31.2142		
Total	68,712.453	119			

Notes: asterisks indicate significant correlations at * *p* < 0.05 and ** *p* < 0.01.

**Table 2:** The split-plot analysis of variance of the four antioxidant enzymes in the muscle tissue of the large yellow croaker at five temperature gradients under stress from natural water cooling.

Source of Variation	Sum of Square	Degrees of Freedom	Mean Square	*F*-Value	*p*-Value
Blocks (replicates)	3.31	5	0.662		
Temperature	76.8437	4	19.2109	65.546 **	<0.001
Main-plot error	5.8618	20	0.2931		
Antioxidant factor	1580.4493	3	526.8164	1259.117 **	<0.001
Temperature × antioxidant factor	156.9203	12	13.0767	31.254 **	<0.001
Split-plot error	31.3801	75	0.4184		
Total	1854.7651	119			

Note: asterisks indicate significant associations at ** *p* < 0.01.

**Table 3:** The split-plot analysis of variance of the six serum enzymes of the large yellow croaker at five temperature gradients under stress from natural water cooling.

Source of Variation	Sum of Square	Degrees of Freedom	Mean Square	*F*-Value	*p*-Value
Blocks (replicates)	65.5821	5	13.1164		
Temperature	2258.2031	4	564.5508	47.63 **	<0.001
Main-plot error	237.0588	20	11.8529		
Serum enzyme	100,879.87	5	20,175.974	1170.82 **	<0.001
Temperature × serum enzyme	7044.8701	20	352.2435	20.441 **	<0.001
Split-plot error	2154.0429	125	17.2323		
Total	112,639.63	179			

Note: asterisks indicate significant correlations at ** *p* < 0.01.

**Table 4:** The AMMI analysis of the antioxidant enzymes in the liver tissue of the large yellow croaker under stress from natural water cooling.

Source of Variation	*df*	SS	MS	*F*	Prob.	% of Total SS
Total	119	68,712.453	577.4156			
Treatment	19	65,577.679	3451.4568	110.1022 **	0	
Gene	3	64,644.578	21,548.193	687.3921 **	0	94.0799
Temperature	4	215.4319	53.858	1.7181	0.1519	0.3135
Interaction	12	717.6689	59.8057	1.9078 *	0.0420	1.0445
IPCA1	6	713.59043	118.93174	3.79395 **	0.0019	99.4317
IPCA2	4	2.89303	0.72326	0.02307	0.9989	0.4031
Residual	2	1.18546	0.59273			
Error	100	3134.7745	31.34774			

Notes: 1. *df*: degrees of freedom; MS: mean squares; SS: sum of squares. 2. **: significant at the 1% probability level, *: significant at the 5% probability level.

**Table 5:** Results of the AMMI analysis of the antioxidant enzymes in the muscle tissue of the large yellow croaker under stress from natural water cooling.

Source of Variation	*df*	SS	MS	*F*	Prob.	% of Total SS
Total	119	1854.7651	15.5863			
Treatment	19	1814.2133	95.4849	235.4638 **	0	
Gene	3	1580.4493	526.8164	1299.1182 **	0	85.2102
Temperature	4	76.8437	19.2109	47.3737 **	0	4.1430
Interaction	12	156.9203	13.0767	32.2468 **	0	8.4604
IPCA1	6	156.70159	26.11693	64.4038 **	0	99.8606
IPCA2	4	0.17806	0.04451	0.1098	0.9788	0.1135
Residual	2	0.04063	0.02031			
Error	100	40.55185	0.40552			

Notes: 1. *df*: degrees of freedom; SS: sum of squares; MS: mean squares. 2. **: significant at the 1% probability level.

**Table 6:** The results of the AMMI analysis of the serum enzymes of the large yellow croaker under stress from natural water cooling.

Source of Variation	*df*	SS	MS	*F*	Prob.	% of Total SS
Total	179	112,639.63	629.2717			
Treatment	29	110,182.94	3799.4118	231.9842 **	0	
Gene	5	100,879.87	20,175.974	1231.903 **	0	89.5598
Temperature	4	2258.2031	564.5508	34.4703 **	0	2.0048
Interaction	20	7044.8701	352.2435	21.5073 **	0	6.2543
IPCA1	8	6894.0632	861.75791	52.6171 **	0	97.8593
IPCA2	6	113.46768	18.91128	1.15468 **	0.3338	1.6106
Residual	6	37.33918	6.2232			
Error	150	2456.6838	16.37789			

Notes: 1. *df*: degrees of freedom; SS: sum of squares; MS: mean squares. 2. **: significant at the 1% probability level.

## Data Availability

Data is contained within the article and Supplementary Material.

## Supplementary Materials

The following supporting information can be downloaded at https://www.mdpi.com/article/10.3390/antiox14101260/s1; Supplementary Material Section SA: Tables S1 and S2: Activities of antioxidant enzymes in liver and muscle of large yellow croaker in different temperature; Table S3: Activities of serum enzymes in blood of large yellow croaker in different temperature; Tables S4 and S5: GGE biplot analysis results of antioxidant enzymes under different natural water cooling stress in liver and muscle; Table S6: GGE biplot analysis results of serum enzymes under different natural water cooling stress in blood; Supplementary Material Section SB: a detailed explanation of the GGE biplot views.
