# Supplementary material for: Genetic Basis of the Antioxidant and Serum Enzyme Activities of the Large Yellow Croaker Larimichthys crocea Under Stress in an Experimental Simulation of Natural Winter Water Cooling
Source: Antioxidants (Basel). 2025 Oct 20;14(10):1260. doi: 10.3390/antiox14101260 (PMC12561848; doi:10.3390/antiox14101260)
Supplement: Supplementary file 1 [file antioxidants-14-01260-s001.zip › antioxidants-3882751-supplementary.pdf]

Supplementary material A

Table S1 Activities of antioxidant enzymes (SOD, POD, CAT and T-AOC) in liver of large yellow croaker in different temperature

Temperature	Antioxidant enzyme	Repeat group 1	Repeat group 2	Repeat group 3	Repeat group 4	Repeat group 5	Repeat group 6
20	SOD/U/mg	53.66	47.59	57.78	32.46	52.93	42.46
20	POD/U/mg	0.97	0.79	1.73	0.88	1.18	0.97
20	CAT/U/mg	4.22	2.98	4.31	4.03	5.11	4.19
20	T-AOC/U/mg	0.62	0.21	0.92	0.75	0.28	0.57
16	SOD/U/mg	53.5	48.91	44.21	33.22	57.73	67.68
16	POD/U/mg	2.19	2.27	1.88	1.93	2.11	2.15
16	CAT/U/mg	4.31	4.69	3.85	3.42	3.71	4.16
16	T-AOC/U/mg	0.94	1.47	0.72	0.96	1.57	0.83
12	SOD/U/mg	76.82	51.21	63.34	53.65	48.91	43.32
12	POD/U/mg	1.44	1.59	1.53	1.24	1.72	1.55
12	CAT/U/mg	3.36	4.71	4.69	3.85	3.42	3.71
12	T-AOC/U/mg	0.38	0.46	0.37	0.37	0.48	0.41
10	SOD/U/mg	58.22	77.61	40.79	61.62	65.78	57.67
10	POD/U/mg	1.16	0.97	1.18	1.26	0.67	1.28
10	CAT/U/mg	3.61	3.77	3.89	4.14	3.66	3.59
10	T-AOC/U/mg	1.03	0.64	0.92	0.94	0.84	0.96
8	SOD/U/mg	64.3	42.5	73	66.5	67.6	61.7
8	POD/U/mg	1.11	0.95	0.89	1.23	1.02	0.83
8	CAT/U/mg	4.14	4.35	4.26	4.86	4.12	4.04
8	T-AOC/U/mg	0.95	0.46	0.37	0.48	0.72	0.46

Table S2 Activities of antioxidant enzymes (SOD, POD, CAT and T-AOC) in muscle of large yellow croaker in different temperature

Temperature	Antioxidant enzyme	Repeat group 1	Repeat group 2	Repeat group 3	Repeat group 4	Repeat group 5	Repeat group 6
20	SOD/U/mg	15.87	11.58	11.34	14.27	13.82	13.29

20	POD/U/mg	0.67	0.79	1.11	1.47	0.8	1.2
20	CAT/U/mg	1.18	1.31	0.89	1.14	1.63	1.03
20	T-AOC/U/mg	0.25	0.38	0.36	0.25	0.27	0.22
16	SOD/U/mg	12.26	8.37	9.29	11.54	12.35	10.38
16	POD/U/mg	0.78	0.95	1.15	0.92	0.88	0.75
16	CAT/U/mg	0.72	1.15	0.83	0.68	0.91	0.86
16	T-AOC/U/mg	0.33	0.45	0.39	0.36	0.37	0.35
12	SOD/U/mg	7.08	8.07	8.02	7.62	8.23	9.14
12	POD/U/mg	0.82	0.66	0.88	0.92	0.76	0.85
12	CAT/U/mg	0.57	0.71	0.78	0.73	0.61	0.73
12	T-AOC/U/mg	0.41	0.29	0.46	0.39	0.33	0.37
10	SOD/U/mg	8.13	6.68	7.17	7.36	9.43	6.18
10	POD/U/mg	0.59	0.66	0.81	0.68	0.67	0.68
10	CAT/U/mg	0.73	0.33	0.45	0.47	0.56	0.68
10	T-AOC/U/mg	0.27	0.18	0.37	0.16	0.14	0.13
8	SOD/U/mg	6.12	5.99	4.43	5.11	5.33	5.13
8	POD/U/mg	0.43	0.64	0.71	0.34	0.28	0.31
8	CAT/U/mg	0.57	0.68	0.44	0.37	0.62	0.41
8	T-AOC/U/mg	0.37	0.29	0.46	0.26	0.35	0.27

Table S3 Activities of serum enzymes (ALT, AST, ALP, LIP, ADA, and GGT) in blood of large yellow croaker in different temperature

Temperature	Serum enzyme	Repeat group 1	Repeat group 2	Repeat group 3	Repeat group 4	Repeat group 5	Repeat group 6
20	ALT /U/L	23.12	22.86	24.79	19.67	24.55	20.14
20	AST /U/mg	100.22	86.07	111.63	126.97	93.15	89.59
20	ALP/U/mg	10.58	14.88	11.2	10.5	12.35	16.49
20	LIP/U/mg	10.52	9.27	7.25	5.63	7.85	8.51
20	ADA/U/mg	3.92	5.31	3.26	4.44	3.17	4.25
20	GGT/U/mg	1.24	1.45	1.57	1.93	1.49	1.26
16	ALT /U/L	21.53	16.22	23.76	22.6	20.79	14.31
16	AST /U/mg	82.09	79.43	67.87	59.25	65.28	75.51
16	ALP/U/mg	10.03	13.66	11.31	9.17	13.16	12.24
16	LIP/U/mg	7.39	9.27	8.99	10.29	8.48	7.05

16	ADA/U/mg	4.61	5.34	4.75	5.15	3.92	3.62
16	GGT/U/mg	1.79	1.81	2.34	2.56	1.42	2.31
12	ALT /U/L	13.65	15.07	17.75	13.91	13.91	17.04
12	AST /U/mg	66.29	65.32	71.23	75.4	82.49	63.52
12	ALP/U/mg	6.68	6.08	8.35	6.55	8.05	6.27
12	LIP/U/mg	10.46	8.09	7.96	11.86	9.19	9.15
12	ADA/U/mg	4.78	5.14	5.08	5.44	6.58	5.53
12	GGT/U/mg	1.97	1.79	1.54	2.24	2.55	2.51
10	ALT /U/L	18.18	16.54	17.22	15.42	16.12	14.87
10	AST /U/mg	53.77	52.86	62.93	58.46	50.15	60.15
10	ALP/U/mg	3.69	3.29	6.81	3.17	4.67	2.95
10	LIP/U/mg	11.79	7.26	10.53	12.89	8.65	7.96
10	ADA/U/mg	5.06	5.69	5.91	5.58	4.25	6.12
10	GGT/U/mg	2.42	2.42	2.31	2.72	2.02	2.02
8	ALT /U/L	16.08	15.16	16.35	14.71	14.34	13.38
8	AST /U/mg	59.52	58.72	54.23	50.66	43.5	51.96
8	ALP/U/mg	4.12	9.16	9.56	4.57	5.35	5.82
8	LIP/U/mg	5.77	7.78	9.28	6.58	10.98	8.55
8	ADA/U/mg	6.45	5.76	4.28	6.49	4.22	5.14
8	GGT/U/mg	3.03	2.17	2.07	2.59	2.28	3.72

Table S4. GGE biplot analysis results of antioxidant enzymes under different natural water cooling stress in liver

**Antioxidant enzymes/ Temperatures**	**Mean activities**	**Deviation**	**PCA1**	**PCA2**	**PCA3**	**Distance from center point (D_i)**
SOD	55.5557	40.1426	8.8348	-0.0405	-0.0016	8.8349
POD	1.3557	-14.0574	-3.0948	-0.2571	0.5845	3.1599
CAT	4.0383	-11.3747	-2.5066	0.7211	-0.0844	2.6096
T-AOC	0.7027	-14.7104	-3.2334	-0.4235	-0.4984	3.2989
20°C	13.3996	-2.0135	3.8984	0.6965	-0.1183	3.9619
16°C	14.5171	-0.8960	4.1145	-0.1994	0.4334	4.1421
12°C	15.5221	0.1090	4.6054	0.0197	0.4231	4.6248
10°C	16.5083	1.0953	4.9524	-0.4851	-0.3765	4.9903
8°C	17.1183	1.7053	5.1484	0.0811	-0.2731	5.1562

Table S5. GGE biplot analysis results of antioxidant enzymes under different natural water cooling stress in muscle

**Antioxidant enzymes/**	**Mean**	**Deviation**	**PCA1**	**PCA2**	**PCA3**	**Distance from**

Temperatures	activities					center point (D_i)
SOD	8.9860	6.2778	3.5673	0.0093	0.0147	3.5673
POD	0.7720	-1.9362	-1.1018	0.2098	-0.2450	1.1481
CAT	0.7590	-1.9493	-1.0985	-0.3777	-0.0275	1.1619
T-AOC	0.3160	-2.3923	-1.3669	0.1586	0.2578	1.4001
20℃	3.9633	1.2551	2.6358	-0.3354	-0.0732	2.6580
16℃	3.2092	0.5009	2.0988	0.1153	0.0643	2.1030
12℃	2.4762	-0.2320	1.5555	0.2122	-0.0333	1.5703
10℃	2.2296	-0.4787	1.4752	0.2023	-0.1430	1.4958
8℃	1.6629	-1.0453	1.0327	0.0130	0.3105	1.0785

Table S6. GGE biplot analysis results of serum enzymes under different natural water cooling stress in blood

Antioxidant enzymes/ Temperatures	Mean activities	Deviation	PCA1	PCA2	PCA3	Distance from center point (D_i)
ALT	17.8013	-0.9819	0.2318	0.2797	1.5968	1.6376
AST	70.6073	51.8241	-10.3485	-0.0080	-0.3302	10.3537
ALP	8.3570	-10.4262	1.9914	-2.0542	0.0233	2.8611
LIP	8.8410	-9.9422	2.0428	1.2889	-0.1209	2.4184
ADA	4.9747	-13.8086	2.7771	0.6071	-0.5249	2.8907
GGT	2.1180	-16.6652	3.3054	-0.1136	-0.6441	3.3695
20℃	25.0300	6.2468	-7.3527	-1.2141	-0.3747	7.4616
16℃	19.7028	0.9196	-5.0479	-0.8254	1.0750	5.2267
12℃	18.3172	-0.4660	-5.0250	0.7192	-1.2550	5.2290
10℃	15.7458	-3.0374	-3.9478	1.8056	0.6283	4.3863
8℃	15.1203	-3.6629	-3.6801	0.6389	0.3138	3.7483

Supplementary material B

The GGE biplots of the relationship among different temperatures (Figure 1-A, Figure 2-A Figure 3-A) mainly analyze the similarity of antioxidant properties/serum enzymes evaluation among temperatures. The included angle of the two line segments indicates the correlation of the activity ranking of the measured antioxidant properties/serum enzymes under the temperature

represented by two line segments. When the included angle of the two segments is an acute angle, the activity ranking of antioxidant properties/serum enzymes under the two temperatures has a positive correlation. The smaller the angle and the higher the correlation, the closer the activity of antioxidant properties/serum enzymes. When the angle between the two segments is an obtuse angle, the activityranking of antioxidant properties/serum enzymes is negatively correlated under the two temperatures. The length of the line segment is the ability of the temperature to distinguish antioxidant factors. The longer the line segment, the stronger the ability to distinguish. The “which-won-where” view of the GGE biplot (Figure 1-B, Figure 2-B and Figure 3-B) divides the experimental regions according to the interaction between antioxidant property/serum enzyme and temperature and reveals the antioxidant property/serum enzyme with the highest activity level in each region. The antioxidant property/serum enzyme located on the top corner of the polygon in each region is the antioxidant property/serum enzyme with the highest activity in this region. The “high activity and activity stability” view of the GGE biplot (Figure 1-C, Figure 2-C and Figure 3-C) can determine the antioxidant property/serum enzyme with high and stable activity. The direction of the transverse oblique line to the right is the approximate average activities of antioxidant properties/serum enzymes in all temperatures. The straight line perpendicular to the transverse slash represents the tendency of antioxidant property/serum enzyme × temperature interaction. The more deviated from the transverse oblique line, the more unstable the vertical line. A GGE biplot with concentric circles view (Figure 1-D, Figure 2-D and Figure 3-D) judges the high activity and activity stability based on the distance from various antioxidant properties/serum enzymes to the central point of antioxidant properties/serum enzymes. The smaller the distance, the higher and more stable the activities of antioxidant properties/serum enzymes.
